# MUNDO: protein function prediction embedded in a multispecies world

**DOI:** 10.1093/bioadv/vbab025

**Published:** 2021-09-29

**Authors:** Victor Arsenescu, Kapil Devkota, Mert Erden, Polina Shpilker, Matthew Werenski, Lenore J Cowen

**Affiliations:** Department of Computer Science, Tufts University, Medford, MA 02155, USA

## Abstract

**Motivation:**

Leveraging cross-species information in protein function prediction can add significant power to network-based protein function prediction methods, because so much functional information is conserved across at least close scales of evolution. We introduce MUNDO, a new cross-species co-embedding method that combines a single-network embedding method with a co-embedding method to predict functional annotations in a target species, leveraging also functional annotations in a model species network.

**Results:**

Across a wide range of parameter choices, MUNDO performs best at predicting annotations in the mouse network, when trained on mouse and human protein–protein interaction (PPI) networks, in the human network, when trained on human and mouse PPIs, and in Baker’s yeast, when trained on Fission and Baker’s yeast, as compared to competitor methods. MUNDO also outperforms all the cross-species methods when predicting in Fission yeast when trained on Fission and Baker’s yeast; however, in this single case, discarding the information from the other species and using annotations from the Fission yeast network alone usually performs best.

**Availability and implementation:**

All code is available and can be accessed here: github.com/v0rtex20k/MUNDO.

**Supplementary information:**

Supplementary data are available at *Bioinformatics Advances* online. Additional experimental results are on our github site.

## 1 Introduction

A great many sophisticated and effective algorithms have been developed, based on techniques such as network propagation, to infer function from protein–protein interaction (PPI) networks (Can *et al.*, 2005; Cao *et al.*, 2013, 2014; Chua *et al.*, 2006; Cowen *et al.*, 2017; Deng *et al.*, 2002; Letovsky and Kasif, 2003; Nabieva *et al.*, 2005; Voevodski *et al.*, 2009). Many of these function prediction methods focus solely on the network of interactions within a single species, using the interconnectivity pattern of PPI data from that species alone to predict functional labels of proteins of unknown function. One large class of such methods that have recently gained a lot of attention are embedding methods. Embedding methods transform the network topology from a graph into a similarity measure between nodes in a vector space, such that the space around a given node is likely to be enriched for nodes of the same or related function (Choobdar *et al.*, 2019; Grover and Leskovec, 2016; Nelson *et al.*, 2019). For example, Cao *et al.* (2013) use a network-propagation measure, diffusion state distance (DSD) to embed the network, and then perform a simple *k*-nearest neighbor algorithm to arrive at its functional predictions. Alternative embeddings, and alternative, more sophisticated classifiers to assign protein function based on the embedded space (see e.g. Grover and Leskovec, 2016) have also been used.

Until recently, these so-called embedding methods were only designed to leverage known functional label information from a single species. However, by recognizing the course of evolution, one can use homology between orthologous proteins in related species to provide additional information when inferring function (Altschul *et al.*, 1990; Fan *et al.*, 2019; Hamp *et al.*, 2013; Loewenstein *et al.*, 2009; Radivojac *et al.*, 2013; Singh *et al.*, 2008). Indeed, the most popular and common way to predict the function of an unknown protein is to BLAST (Altschul *et al.*, 1990; McGinnis and Madden, 2004) its sequence against the database of all sequenced proteins in multiple species and transfer functional annotation from its closest annotated match to predict its function. This method leverages the vast power of evolution, but ignores network information entirely. Recently, Fan *et al.* (2019) introduced MUNK, a novel co-embedding method to transfer functional annotations between multiple species. By treating some of the easiest to recognize orthologous proteins as *landmarks*, identifying them and mapping them together in the embedded space, MUNK is able to *co-embed* multiple PPI networks into the same vector space, combining the power of single-network embedding methods with the possibility of transferring annotation across species.

**Fig. 1.**
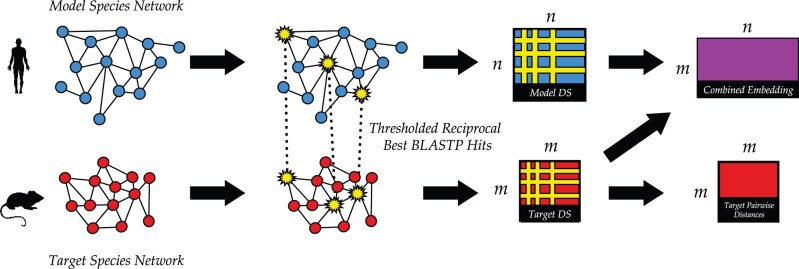
Overview of each step of the MUNDO algorithm, when leveraging the human PPI network to better predict functional annotations in mouse. The human (blue) and mouse (red) PPI networks are given. The red square represents the DSD single-network embedding of the mouse network, and the purple rectangle represents the MUNK co-embedding of the mouse and human networks. MUNDO assigns the functional label of a protein of unknown function based on a weighted vote of its *c* closest MUNK neighbors and its *d* closest DSD neighbors. The landmarks are represented by the corresponding yellow stars in each network. The method of using thresholded reciprocal best BLASTP hits to determine the landmarks for the MUNK embedding is described in Section 3.2. Note that only a relatively small subset of the nodes in each network are paired with nodes in the other, since many nodes’ best hits do not meet our stringent thresholds

**Fig. 2.**
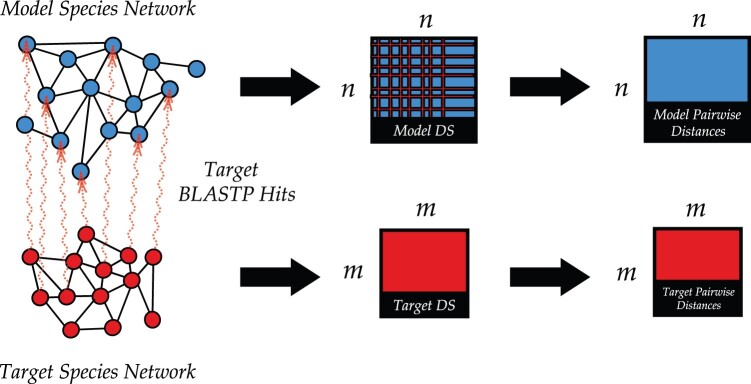
Simple homology transfer methods. For the alternative simple methods that incorporate homology that we also compare to the two network embeddings are single-network embeddings. In the model (blue) species, the neighborhood of the top BLAST hit in the model (blue) network to the target protein in the target network is combined (in six different proposed methods) with its neighborhood in the target network (red). A DS embedding for each network is then used to generate a pairwise DSD distance matrix for each network. BLAST hits are represented by the connections between red and blue nodes in the leftmost diagram

In this paper, we study how best to utilize the power of co-embeddings to predict GO (the gene ontology) (Botstein *et al.*, 2000) functional label annotations in one (possibly more sparsely annotated) species by also using the annotations and interactions in a related (possibly better annotated) species. To test our methods, we mimic the MUNK paper and consider the same pairs of species: *Mus musculus* (mouse) and *HomoSapiens* (human), and the two yeast species *Saccharomycescerevisiae* and *Schizosaccharomycespombe*. We introduce MUNDO, a method that simultaneously combines functional predictions derived from the single PPI network in the target species with functional predictions derived from the hybrid embedding; currently, we use the MUNK embedding directly as our co-embedding for MUNDO (Fig. 1). We tune parameters that determine the relative weight that is given to the function information in the single and hybrid networks, and demonstrate a large range of settings for which MUNDO improves on MUNK alone. We also compare MUNK and MUNDO to a range of simpler baseline approaches that transfer information between the two protein networks on a protein-by-protein basis (Fig. 2). In all four cospecies experiments (mouse/human, human/mouse, bakers/fission, fission/bakers) we find that MUNDO performs better than MUNK and the other baseline multispecies approaches. In three of the four experiments, MUNDO has the best mean predictive accuracy; in the fourth (bakers/fission), the single-network DSD method of Cao *et al.* (2013) which discards all cross-species information outperforms all the tested methods that try to leverage it except MUNDO itself when the training data is the sparsest (only 1/10 of the labels used for training). Finally, we discuss the settings in which MUNDO can be advantageously deployed.

### 1.1 Algorithm overview

As shown above, in Figure 1, MUNDO is concerned with two separate embeddings: a single-network embedding and a combined embedding. The single-network embedding (red box in Fig. 1) encodes the relationships between nodes in the target network alone. MUNDO uses the DSD metric introduced by Cao *et al.* (2013) for its single-network embedding function prediction method (Section 2.1) The conetwork embedding (purple box in Fig. 1), is the MUNK embedding from Fan *et al.* (2019). The input to MUNK is a set of corresponding *landmarks* that are identified to create the co-embedding (yellow starred nodes in Fig. 1). We describe the method we use to choose landmarks in Section 3.2 and then describe the resulting MUNK co-embedding component of MUNDO in Section 2.5.1.

#### 1.1.1 Single-network embedding

The target species PPI network is embedded using the DSD metric introduced by Cao *et al.* (2013). DSD captures a fine-grained representation of the local topology of a network through a series of random walks and tends to reduce the influence of hub nodes. A detailed review of DSD is presented in Section 2.1.

#### 1.1.2 Multinetwork embedding

The MUNK algorithm by Fan *et al.* (2019) produces a combined embedding of the model and target species when given a subset of protein pairs to be identified across the networks to act as *landmarks* to join the two networks. We identify landmarks by searching for the reciprocal best BLAST hits, the recommended method from *Fan et al.*’s paper. The combined embedding captures the relative similarities between nodes across the two networks, and is based on Fan *et al.*’s MUNK algorithm which hinged on the use of homologs as *landmarks* to transfer functional information between two PPI networks. The method we use to select MUNK landmarks is described in Section 3.2 and a detailed review of the MUNK co-embedding follows in Section 2.2.

#### 1.1.3 MUNDO label assignment

Once the DSD and MUNK embeddings are constructed, MUNDO employs a variation of the simple majority vote algorithm (Schwikowski *et al.*, 2000). MUNDO then predicts a GO functional label for each node based on a weighted majority vote among the *c* closest combined network and *d* closest DSD single-network neighbors in the embedded spaces.

### 1.2 Paper outline

The remainder of this paper is structured as follows: Section 2.1 gives a detailed review of DSD and Section 2.2 gives a detailed review of MUNK. Section 2.3 presents six different alternative methods (in a direct, more simplistic way than MUNK or MUNDO) to predict function taking homology relationships between proteins in the two species into account. Section 2.4 explores different parameter settings for how *many* neighbors in each of the networks should be voting, as well as how heavily weighted the votes from one network should be compared to the other. In MUNDO and the other methods, we find a large range of parameters where the good performance of MUNDO is quite robust (Section 4 and Supplementary Tables). Section 4 compares the performance of MUNDO against standalone DSD (a single-network method that does not use cross-species information), MUNK, and the simple cross-species methods. We show that in every case, MUNDO outperforms MUNK and the other cross-species methods in a stringent cross-validation experiment. Furthermore, the margin of superiority of MUNDO’s performance increases as the size of the available training set decreases (Section 4). In all but one case, MUNDO is the best performer overall; in one case (Baker’s yeast as the model species and Fission yeast as the target species), we find the DSD single-network method outperforms all the cross-species methods, including MUNDO. Finally, we discuss the evolutionary distance between species where an MUNDO approach is currently feasible, and possible extensions to more distant species in Section 5.

## 2 Methods

### 2.1 DSD overview

We review the DSD distance from (Cao *et al*., 2013). Consider the undirected graph *G*(*V*, *E*) on the vertex set $V=\{v_{1},v_{2},v_{3},\ldots ,v_{n}\}$ and |V|=n. We define $He^{\left\{t\right\}}(A,B)$ to be the expected number of times that a random walk starting at node *A* and proceeding for *t* steps, will visit node *B*. In what follows, assume *t* is fixed, and when there is no ambiguity in the value of *t*, we will denote $He^{\left\{t\right\}}(A,B)$ by *He*(*A*, *B*).

We define the *n*-dimensional diffusion state (DS) vector $He(v_{i}),\;\forall v_{i}\in V$, where

(1) $$He(v_{i})=(He(v_{i},v_{1}),He(v_{i},v_{2}),\ldots ,He(v_{i},v_{n})).$$

Then the DSD between two vertices *u* and *v*, ∀u,v∈V is defined as

(2) $$\mathrm{DSD}(u,v)=|\left|He\left(u\right)-He\left(v\right)\right|{|}_{1},$$

where $||He(u)-He(v)|{|}_{1}$ denotes the *l*_1_ norm of the *He* vectors of *u* and *v*.

In Cao *et al.* (2013), it is proved that DSD is a metric (including satisfying triangle inequality), and furthermore, that DSD converges as the *t* in $He^{\left\{t\right\}}(A,B)$ goes to infinity, allowing us to define DSD independent from the value *t*.

### 2.2 Munk overview

MUNK (Fan *et al*., 2019) consider a model network $G_{1}=(V_{1},E_{1})$ and a target network $G_{2}=(V_{2},E_{2})$ with $|V_{1}|=m$ and $|V_{2}|=n$. MUNK requires in addition *landmark sets L*_1_ and *L*_2_ to be specified, with $L_{1}\subset V_{1}$ and $L_{2}\subset V_{2}$, with $\left|L_{1}|=|L_{2}\right|$, and a bijection between them. We discuss how *L*_1_, *L*_2_ are chosen and how the associated bijection is constructed below.

MUNK constructs kernel (similarity) matrices $D_{1}\in R^{m\times m}$ and $D_{2}\in R^{n\times n}$ corresponding to *G*_1_ and *G*_2_. Next, it constructs the Reproducing Kernel Hilbert Space (RKHS) vector representations *C*_1_ for nodes in the model network *G*_1_ from the factorization $D_{1}=C_{1}C_{1}^{T}$. Let $C_{1L}$ be the subset of the rows of *C*_1_ corresponding to landmarks, and let $D_{2L}$ be the subset of the rows of *D*_2_ corresponding to landmarks (in corresponding order). The key step then is to construct the vector representations of the nodes in the target network *G*_2_. To do this, we treat the similarity scores $D_{2L}$ in the target network as if they applied to the corresponding landmarks in the model network *G*_1_. For a given node in the target network, we want to find a vector for the node such that its inner product with each model landmark vector is equal to its diffusion score to the corresponding target landmark. This implies that the RKHS vectors, ${\hat{C}}_{2}$, for nodes in the target network *G*_2_ should satisfy $D_{2L}=C_{1L}{\hat{C}}_{2}^{T}$. This under-determined linear system has solution set

(3) $${\hat{C}}_{2}^{T}={\hat{C}}_{1L}^{†}D_{2L}+(I-{\hat{C}}_{1L}^{†}C_{1L})W,$$

where ${\hat{C}}_{1L}^{†}$ is the Moore–Penrose pseudoinverse of $C_{1L}$, and *W* is an arbitrary matrix. We choose the solution corresponding to *W* = 0, meaning that the vectors ${\hat{C}}_{2}^{T}$ are the solutions having minimum norm.

The resulting solution, ${\hat{C}}_{2}$, represents the embedding of the nodes of *G*_2_ (the target) into the same space as the nodes of *G*_1_ (the model). We can then compute similarity scores for all pairs of nodes across the two networks as $D_{12}=C_{1}{\hat{C}}_{2}^{T}$. This yields *D*_12_, an *m* × *n* matrix of similarity scores between nodes in the model and target networks. *D*_12_ is shown in Figure 1 as the ‘combined embedding’ (purple).

#### 2.2.1 Landmark set construction

In order to finish specifying the MUNK co-embedding, it is necessary to describe how the landmark sets and landmark bijection is constructed. The set of landmarks is based on *reciprocal best BLAST hits*: for each node *u* in the target network, we BLAST *u* (using BLASTP with a BLOSUM62 matrix and word size 3) against all nodes *w* in the model network, and for each node *v* in the model network, we BLAST *v* against all nodes *z* in the target network. A pair of nodes (*u*, *v*) with *u* in the target network and *v* in the model network for which *u*’s best BLASTP hit is *v* and *v*’s best BLASTP hit is *u* is called a reciprocal best BLAST hit. We set thresholds of percent query coverage and percent sequence identity and keep all the reciprocal best BLAST hits that meet these thresholds as landmarks for MUNK (or the MUNK portion of MUNDO) (Section 2.4.1 for how we set these parameters in our experiments).

### 2.3 Simple homology transfer methods

In addition to the MUNK co-embedding method, and the DSD single-network method, we also compare MUNDO to six different simple homology transfer methods that use top BLAST hits to transfer functional information between networks in a direct point-to-point fashion. For all six methods: for each node *u* in the target network, we BLAST *u* (using BLASTP with a BLOSUM62 matrix and word size 3) against all nodes in the model network. A DS embedding is computed for each network individually, just as in the single-network approach discussed above. Finally, pairwise distance matrices for the target and model networks are generated from each embedding matrix. These are depicted above in Figure 2 as the final blue and red matrices, respectively.

Let *u* refer to the node in the target network whose function we are trying to predict:

1. **Parallel** **network** **top** **blast** **hit**: The predicted function for node *u* is determined by a majority vote amongst its *d* nearest neighbors in the target network along with *u*’s top BLAST hit *v* in the model network. Each vote from *u*’s *d* nearest neighbors is weighted by a factor of *α*, to *v*’s single vote.
2. **Parallel** **network** **top** **blast** **hit + neighborhood**: The predicted function for node *u* is determined by a majority vote amongst its *d* nearest neighbors in the target network, *u*’s top BLAST hit *v* in the model network, and the *c* nearest neighbors *of the top BLAST hit v* in the *model* network. Each vote from *u*’s *d* nearest neighbors is weighted by a factor of *α*, compared to *v*’s single vote along with the votes of its *c* nearest neighbors.
3. **Parallel** **network** **all** **blast** **hits**: The predicted function for node *u* is determined by a majority vote amongst its *d* nearest neighbors in the target network and *u*’s first 100 BLAST hits (the default number of hits returned by BLAST). Each vote from *u*’s *d* nearest neighbors is weighted by *α*, compared to each of the 100 BLAST hit votes.
4. **Parallel** **network** **all** **blast** **hits + neighborhoods**: The predicted function for node *u* is determined by a majority vote amongst its *d* nearest neighbors in the target network and *u*’s first 100 BLAST hits (the default number of hits returned by BLAST). Each of the unfiltered one hundred BLAST hits’ *c* nearest neighbors in the *model* network are also included in the vote. Each vote from *u*’s *d* nearest neighbors is weighted by *α*, compared to each of the 100 BLAST hit votes and the votes of each hit’s *c* nearest neighbors.
5. **Parallel** **network** **thresholded** **blast** **hits**: The predicted function for node *u* is determined by a majority vote amongst its *d* nearest neighbors in the target network and all of *u*’s BLAST hits that meet a minimum threshold for query coverage and percent identity are included in the vote. In this case, p=85% percent id and q=90% query coverage thresholds were used. Each vote from *u*’s *d* nearest neighbors is weighted by *α*, compared to each of the thresholded BLAST hits’ votes.
6. **Parallel** **network** **thresholded** **blast** **hits + neighborhoods**: The predicted function for node *u* is determined by a majority vote amongst its *d* nearest neighbors in the target network and all of *u*’s BLAST hits that meet a minimum threshold for query coverage and percent identity are included in the vote. Furthermore, each of the one thresholded BLAST hits’ *d* nearest neighbors in the *model* network are included. Once again, p=85% percent id and q=90% query coverage thresholds were used. Each vote from *u*’s *d* nearest neighbors is weighted by *α*, compared to each of the thresholded BLAST hits’ votes and the votes of each hit’s *c* nearest neighbors.

### 2.4 MUNDO and competing methods parameters

MUNDO needs to set five parameters: *p*, *q*, *c*, *d* and *α* where *p* and *q* concern the quality of the RBH landmarks (for MUNDO and also MUNK; for the simple homology methods they are a threshold on the quality of the ordinary BLAST hits as described in Section 2.3), and thus effect the embeddings, but *c*, *d*, *α* only effect which neighbors vote with what weight. MUNDO, and all the methods we compare MUNDO to, except MUNK, set a parameter *d*, that represents the size of the DSD neighborhood in the target species network that is considered. MUNDO and MUNK also set a parameter *c*, which controls the size of the DSD neighborhood in the combined embedding—some of the other competitor methods consider network neighborhood in the source network, and for these we also term the parameter that controls the size of this network neighborhood *c*.

#### 2.4.1 Reciprocal BLAST hit landmark quality

Our setting of *p* and *q* ensures that the landmarks are strong reciprocal best BLAST hits (RBH) for the human/mouse network co-embedding in both MUNK and MUNDO. We require that the *coverage* (defined as the proportion of the sequence BLAST aligns) is at least 90% and the *percent sequence identity* is at least 85%. This gives us 309 landmarks between human and mouse. This matches the recommended size and quality of the landmark set in Fan *et al.* (2019). For the two yeast networks, because of the large evolutionary distance between *S.cerevisiae* and *S.pombe*, keeping the same *p* and *q* thresholds would only result in eight landmarks. To increase the number of landmarks it is necessary to relax the thresholds on reciprocal BLAST hits. However, as the thresholds are lowered, the amount of noise in the co-embedding increases, and predictive accuracy is negatively impacted. Therefore, we searched for settings that would keep the percent coverage and sequence identity reasonably high, while giving us at least 60 landmarks (where 60 was chosen to match the mouse/human mapping since the number of proteins in the yeast network is roughly a fifth the size of the number of proteins in our human PPI network—network statistics appear in Section 3.1). We ended up choosing (*q*, *p*) = (75%, 50%), resulting in 61 landmarks. [We also tried an embedding based on the 79 landmarks obtained with (*q*, *p*) set to (50%, 50%), and found that performance was very similar—see Supplementary Materials].

#### 2.4.2 Majority vote parameters

In order to explore the remaining method parameters *d*, *c* and *α* in a principled way, we randomly split the labeled nodes 50/50 for the first species co-embeddings experiment (human as model species; mouse as target) into separate training and validation sets. Parameters were then explored only on the training set, which was further split into 80% training and 20% testing folds for standard 5-fold cross-validation experiments (Fig. 4). We first look at the effect of varying the *c* and *d* parameters in *all* methods (noting DSD rewrites *c* = 0, and MUNK rewrites *d* = 0), by first setting *α* to be 1.0 in MUNDO, so that votes from the *d* closest proteins in the individual and *c* closest neighbors in the combined network had equal weight votes. Supplementary Table S1 gives the results of a grid search over the parameters {5,10,20} for both *c* and *d* on the training set in 5-fold cross-validation. As can be seen in Supplementary Table S1, over MUNDO and all competitor methods, we find that setting *d* = 20 for the number of neighbors in the target species outperforms all other *d* settings (note that MUNK does not set the *d* parameter, only *c*).

We also explored whether weighting votes differently according to whether they came from the *d* target species neighbors or the *c* combined species network neighbors would matter. Supplementary Table S2 explores different settings for *α*, the relative weight that is given to votes from functional labels in the target versus combined networks. Based on this performance, we recommend default MUNDO parameters of *d* = 20, and α=1.5 for human–mouse. We report the performance of MUNDO and all competitor methods over all three *c* choices in our independent validation set in Table 1 of our main results. As can be seen in Table 1 and Supplementary Tables S1–S4, MUNDO results were relatively robust to different *c* values, but *c* = 10 performs slightly better than other settings. We then keep these same parameter values (i.e. d=20,c=10,α=1.5) for our mouse/human and both yeast/yeast experiments.

**Table 1.** Mean percent accuracy, *F*1-max and Resnik scores reported for *H.sapiens* → *M.Musculus*

	DSD	Top blast hit	Top blast hit + neighbors	All blast hits	All blast hits + neighbors	Thresholded blast hits	Thresholded blast hits + neighbors	MUNK	MUNDO
(d,c)=(20,5)									
Accuracy	13.23	14.12	14.07	13.46	13.16	13.67	13.69	10.87	**15.05**
*F*1-max	10.03	10.67	10.69	10.20	10.01	10.34	10.42	7.55	**11.25**
MF Resnik	2.71	2.73	2.23	2.63	2.07	2.72	2.67	2.52	**2.97**
BP Resnik	2.11	2.25	2.24	2.25	1.83	2.11	2.45	**3.57**	2.45
(d,c)=(20,10)									
Accuracy	13.23	14.12	13.72	13.46	12.76	13.67	13.58	10.69	**15.12**
*F*1-max	10.03	10.67	10.42	10.20	9.81	10.34	10.40	8.36	**11.41**
MF Resnik	2.71	2.73	1.99	2.63	1.80	2.72	2.63	2.82	**3.17**
BP Resnik	2.11	2.25	2.49	2.25	2.26	2.11	2.71	**3.79**	2.82
(d,c)=(20,20)									
Accuracy	13.23	14.12	12.93	13.46	12.11	13.67	13.42	11.15	**14.42**
*F*1-max	10.03	10.67	9.93	10.20	9.28	10.34	10.27	8.24	**10.87**
MF Resnik	2.71	**2.73**	2.01	2.63	1.93	2.72	2.63	1.94	2.66
BP Resnik	2.11	2.25	2.49	2.25	2.07	2.11	2.59	2.27	**2.76**

*Notes*: The performance of each method is reported over the independent validation set (half the size of the original data set), with the entirety of the training set used for labeled nodes. All competing methods shown with *α* = 1, *d* = 20; and c={5,10,20} where note that only the ‘+ neighbors’ columns, MUNK and MUNDO have a source or combined network in which to set a separate *c* parameter. MUNDO based on Supplementary Table S2 sets α=1.5. MUNDO performs best across the board, except for BP Resnik, where MUNK is often better. Best performing results for each parameter setting across the completing methods are in bold. For results over all tested parameter settings, see Supplementary Materials.

### 2.5 Full details of MUNDO

#### 2.5.1 Co-embedding

We give more technical details for how the MUNK co-embedding is computed. Let *M* be the *n* × *n* matrix consisting of the DS vectors for the model species network and *T* be the *m* × *m* matrix consisting of the DS vectors for the target species network. These matrices are depicted in Figure 1 as the blue and red square matrices, respectively.

Define $\Lambda =\{\lambda_{1},\lambda_{2},\ldots \lambda_{n}\}$ and $ϒ=\{v_{1},v_{2},\ldots ,v_{n}\}$ to be the set of eigenvalues and eigenvectors of the model network *M*, respectively. We use these to compute the model network embedding *N* relative to which *T* will be co-embedded as follows:

(4) $$N=ϒ\cdot \Lambda^{1/2}I_{n}=\left[\begin{array}{ccc} | & & |\\ v_{1} & \ldots & v_{n}\\ | & & | \end{array}\right]\cdot \left[\begin{array}{cccc} \lambda_{1}^{1/2} & 0 & \ldots & 0\\ 0 & \lambda_{2}^{1/2} & \ldots & 0\\ \vdots & \vdots & \ddots & \vdots \\ 0 & 0 & \ldots & \lambda_{n}^{1/2} \end{array}\right]$$.

Define *r_i_* to be the *i*th reciprocal best hit for a given network, and let there be *p* reciprocal best hit pairs in total Let $A_{\left[i\ldots j\right]}$ be a matrix comprised of rows i,i+1,…,j of a given matrix *A*. Finally, let $A^{†}$ denote the pseudoinverse [Moore–Penrose inverse (Barata and Hussein, 2012)] of a given matrix *A*. The target network embedding *C* relative to the model network embedding *N* can therefore be computed as

(5) $$C^{T}={\left(N_{\left[r_{i}\ldots r_{p}\right]}^{T}\right)}^{†}\cdot T_{\left[r_{i}\ldots r_{p}\right]}.$$

In addition to the *m* × *n* combined embedding matrix, *C*, we also compute *P*, an *m* × *m* pairwise distance matrix derived from *T*, the matrix of DS vectors. For each pair of *m*-dimensional vectors $t_{i},t_{j}\in T$, the *l*_1_-norm is computed and stored in *P* as a scalar value $P_{i,j}$. By definition, *P* is necessarily square. Matrices *C* and *P* are shown in Figure 1 as the rightmost purple and red matrices, respectively.

After sorting both output matrices *P* and *M* by distance, we are able to quickly identify the *d* nearest DSD neighbors and *c* nearest combined embedding neighbors of each node *i* in the target network whose function we would like to predict. Respectively, these neighbors can be found at $P_{i,[0\ldots d-1]}$ and $M_{i,[0\ldots c-1]}$.

## 3 Experimental setup

### 3.1 Networks

The human PPI network consists of the 25 672 unique nodes and 487 840 unique interaction edges downloaded from BioGRID version 3.5.188, excluding self-loops. Of the included edges, 479 400 encoded physical interactions and 8440 encoded genetic interactions. Taking the largest connected component yields 17 017 nodes and 407 653 edges.

The mouse PPI network consists of the 15 979 unique nodes and 74 966 unique interaction edges downloaded from BioGRID version 3.5.188, excluding self-loops. Of the included edges, 74 646 encoded physical interactions and 320 encoded genetic interactions. Taking the largest connected component yields 8543 nodes and 45 605 edges.

The *S.cerevisiae* PPI network consists of the 4701 unique nodes and 71 688 unique interaction edges downloaded from BioGRID version 4.2.192, excluding self-loops. Of the included edges, 14 008 encoded physical interactions and 57 680 encoded genetic interactions. Taking the largest connected component yields 4335 nodes and 61 856 edges.

The *S.pombe* PPI network consists of the 7335 unique nodes and 607 030 unique interaction edges downloaded from BioGRID version 4.2.192, excluding self-loops. Of the included edges, 123 802 encoded physical interactions and 483 228 encoded genetic interactions. Taking the largest connected component yields 5817 nodes and 538 250 edges.

### 3.2 Landmark selection

**Fig. 3.**
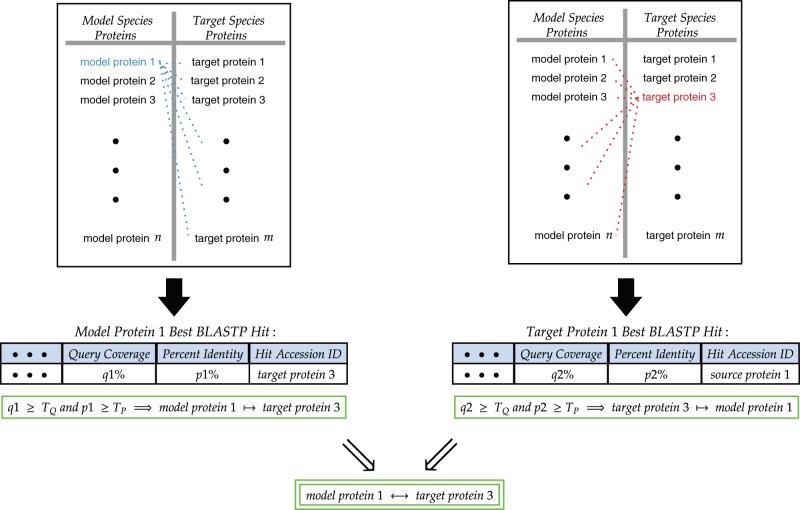
The schematic above details the landmark selection process, beginning with the identification of reciprocal best BLAST hits and ending with their thresholding. Each protein in the target network (shown in red) is BLASTed against all nodes in the model network (shown in blue), and vice versa. A protein from the model species, and its top-ranked BLASTP hit in the target species form a reciprocal best BLASTP hit (RBH) if when the top-ranked BLASTP hit from the target species is BLASTed back against all nodes in the model species, the top-ranked BLASTP hit is the original protein from the target species. RBHs are added to the set of *landmarks* only if the query coverage and percent identity between the two proteins meet certain thresholds, *T_q_* and *T_p_*, which are tunable hyperparameters for both MUNK and MUNDO methods (hereafter referred to as *q* and *p*, respectively)

The quality of the co-embedding performed later in the process depends heavily on the ‘overlap’ between the model and target networks. Traditionally, overlap refers to the set of nodes that exist in both networks. In this case, each network belongs to a distinct species, so no protein can exist in both networks simultaneously. We therefore redefine overlap in this context to refer to the set of reciprocal best BLAST hits between two networks. Any hit which does not meet a minimum similarity threshold set by the user is filtered out. The number of hits can vary greatly between organisms, but experimental results have shown that roughly 300 RBHs are sufficient for a high-quality embedding. This is in line with the minimum number of landmarks recommended in the MUNK (Fan *et al.*, 2019) algorithm. A schematic of the selection process is provided above in Figure 3.

**Fig. 4.**
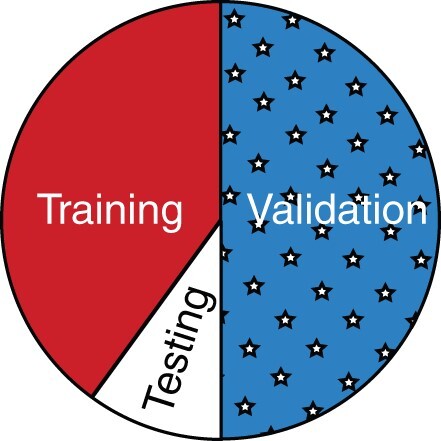
Illustration of the train–test–validation split described in Section 2.4. As shown, the dataset was initially split in half, with 50% being reserved for validation. 5-Fold cross-validation was then performed on the remaining 50% of the data, with 80% of the remaining half being used for training and 20% of the remaining half being used for testing

This voting algorithm allows nodes from a different species’ network to vote alongside the closest neighbors of a given node, each with their own tunable weight hyperparameters. Once the final list of votes F(v) has been compiled, the most frequently appearing vote in the list is used as the ultimate prediction for the function of *v*. Note that this is quite similar to the traditional weighted majority vote algorithm, except each species has its own weight parameter. The optimal values of *α*, setting the weight of the model species votes as compared to the target species votes, are explored in Supplementary Materials.

### 3.3 Functional labels

Functional labels for all species were downloaded from EMBL-EBI’s UniProt GOA database, version 201. We considered GO labels from the biological process (BP) and molecular function (MF) hierarchies. The set of GO terms was filtered to those in an intermediate range of specificity, retaining GO terms that annotate between 50 and 500 nodes in the target network for human ↔ mouse and between 50 and 300 nodes in the target network for *S.pombe* ↔ *S.cerevisiae*.

### 3.4 Performance measures

We measure the performance of the different methods in three ways. The simplest way, percent accuracy only considers the top prediction for each node. The others consider a ranked list of the top three predictions, which in GO are often more or less specific and related terms: the *F*1 score, however, does not explicitly take into account the hierarchical structure of GO, whereas the Resnik score measures the information content in reference to the GO hierarchy.

**Evaluation** **method** **1:** **percent** **accuracy.** This metric simply measures the percent of nodes whose top predicted functional label is correct, meaning it is among the set of true functional labels assigned to that node.

**Evaluation** **method** **2:** **hierarchy** **agnostic** $F1^{*}$ **method.** This evaluation metric, which corresponds to the protein-centric evaluation method in the CAFA challenge (Radivojac *et al.*, 2013; Zhou *et al.*, 2019), scores a multilabel function prediction set, but still ignores the hierarchical nature of the GO annotations while scoring predictions. For a particular protein *i*, let *T_i_* be the set representing its true GO annotation and $P_{i}(\tau )$ represent the set of GO annotations predicted by the function prediction method with likelihood greater than the confidence threshold *τ*. Then, we can compute the precision and recall for the protein *i* at the threshold *τ* as

(6) $${\text{prec}}_{i}(\tau )=\frac{\left|P_{i}(\tau )\cap T_{i}\right|}{\left|P_{i}(\tau )\right|},$$

(7) $${\text{recall}}_{i}(\tau )=\frac{\left|P_{i}(\tau )\cap T_{i}\right|}{\left|T_{i}\right|}.$$

The average precision and recall for a particular confidence threshold *τ* is

(8) $$\text{prec}(\tau )=\frac{1}{M}\sum_{i=1}^{M}{\text{prec}}_{\alpha_{\tau }(i)}(\tau ),$$

(9) $$\text{recall}(\tau )=\frac{1}{N}\sum_{i=1}^{N}{\text{recall}}_{i}(\tau ),$$

where $\alpha_{\tau }$ represents the set of all proteins which have at least one GO annotation predicted at the confidence interval *τ* [$\alpha_{\tau }(i)$ represents its *i*th member], *M* is the size of the set $\alpha_{\tau }(i)$ and *N* is the total number of proteins in the test set.

We can then compute the *F*1 score at confidence *τ*, and $F1^{*}$ as

(10) $$F1(\tau )=2\frac{\text{prec}(\tau )\cdot \text{recall}(\tau )}{\text{prec}(\tau )+\text{recall}(\tau )},$$

(11) $$F1^{*}=\underset{\tau }{\max}F1(\tau ).$$

#### 3.4.1 Evaluation method 3: Resnik similarity metric

This metric models the hierarchical nature of GO by introducing the information content of a GO term (Jiang *et al.*, 2016) in the context of its ancestors. Let ℓ be a GO term and *L* be the subgraph generated by all its ancestor labels, including ℓ. The information content of ℓ is defined formally as

(12) i(ℓ)=−log(Pr(L)),

where the joint probability *Pr*(*L*) is computed as

(13) $$Pr(L)=\prod_{v\in L}Pr(v|P(v)).$$

The term Pr(v|P(v)), *v* being a GO term and P(v) representing the parents of *v*, denotes the probability that we get *v* from P(v) after further ontological specialization. Expression 9 can be further simplified using expression 10 to obtain

(14) $$i(\ell )=-\sum_{v\in L}\;\text{log}\;(Pr(v|P(v)))=\sum_{v\in L}ia(v).$$

The term *ia*(*v*), referred to as information accretion of the annotation *v*, denotes the increase in the information obtained through the addition of child GO term (*v*) to the set of its parent terms (or P(v)).

Resnik similarity (${\text{res}}_{\text{tt}}$) between two GO terms, *x* and *y*, is

(15) $${\text{res}}_{\text{tt}}(x,y)=i(\text{lca}(x,y)),$$

where lca(x,y) represents the least common ancestor between *x* and *y*.

We next extend this similarity measure between GO terms to a similarity metric between two sets of GO terms, using a similar method to Zhao and Wang (2018). Define ${\text{res}}_{\text{st}}(X,y)$, which takes a GO set *X*, and a GO term *y* as

(16) $${\text{res}}_{\text{st}}(X,y)=\underset{x\in X}{\max}{\text{res}}_{\text{tt}}(x,y).$$

Then, the Resnik similarity ${\text{res}}_{\text{ss}}$ for GO sets *X* and *Y* can be defined as

(17) $${\text{res}}_{\text{ss}}(X,Y)=\frac{\sum_{x\in X}{\text{res}}_{\text{st}}(Y,x)+\sum_{y\in Y}{\text{res}}_{\text{st}}(X,y)}{\left|X|+|Y\right|}.$$

Let *Q* be the set containing all the test proteins, *T_q_* be the true GO terms and $P_{q}(\tau )$ be the predicted GO terms at the confidence interval *τ*, for a protein q∈Q. We compute

(18) $$\text{RES}=\underset{\tau }{\max}\frac{1}{\left|Q\right|}\sum_{q\in Q}{\text{res}}_{\text{ss}}(T_{q},P_{q}(\tau )).$$

We measure the Resnik similarity over both the MF and BP hierarchies of GO terms.

### 3.5 Inverted cross-validation experiment

In the most common form of *k*-fold cross-validation, *k* − 1 of the folds are used for *training* and the remaining fold is used for *testing*. Because we were especially interested in performance of function prediction methods when the amount of training data is sparse, we copied the experimental setup of Lazarsfeld *et al*. (2021), which ‘inverts’ the relative sizes of the training and test data, so that, only one of the folds is used for training, and the other *k* − 1 have all their annotations removed and are placed in the testing set. Thus, for our inverted cross-validation experiment, the larger *k* is, the smaller the size of the training set. Mean and standard deviation of percent accuracy, calculated as the percent of time a protein in the test set is assigned a label which is correctly among its true annotations, is computed over five different runs of *k*-fold cross-validation, for k=2,4,6,10 in our experiments. In addition, we compute precision and recall by looking at the top *r* predicted labels (in our case, we present results for *r* = 3) using the method of Deng *et al.* (2004), and report the maximum *F*1 score according to their first recommended method, and also compute Resnik scores for both the MF and BP hierarchies.

## 4 Results

Because in our first species experiment (*human* as the model species and *mouse* as the target species) we needed to tune parameters, we did standard 5-fold cross-validation to tune those parameters for MUNDO and all its competitors, and then presented the results on the independent validation set. As shown in Table 1, MUNDO with parameters set as described in Section 2.4 produces substantially better percent accuracy and *F*1-max scores than DSD, MUNK and all six simple homology methods.

In the other species experiments, we decided to fix MUNDO’s parameters to the recommended defaults based on the first species experiment. This eliminates the need for an independent validation set for these experiments. For these experiments, we therefore decided to run *inverted* cross-validation experiments (Section 3.5 about our unusual design of inverted cross-validation: the 10-fold experiment inverts the usual role of training and test sets: with 1-fold used for training and 9-folds used for test). We compared MUNDO to DSD, MUNK, and the top performing variant of the simple methods in the first experiment, namely top BLAST hit + neighborhood, in 2-fold, 4-fold, 6-fold and 10-fold inverted cross-validation experiments and results appear in Tables 2–4. MUNDO performs best or second best in all three experiments; it is best by most measures when mouse is the model species and human is the target, and when *pombe* is the model species and *cerevisiae* is the target, but is outperformed by single-network DSD in the target species when *cerevisiae* is the model species and *pombe* is the target, except when the size of the training set is set to the smallest size we tested (the 10-fold experiment). Interestingly, we note that the margin of improvement for MUNDO *increases* as the number of proteins that contribute functional labels in the training data *decreases*. Looking at the runner up methods is also interesting: the single-network method is slightly more accurate than MUNK when we reserve half the nodes for the training set; MUNK becomes comparable or very slightly more accurate compared to the single network in the 10-fold experiment when the amount of training data is much lower. Top blast hit + neighborhood (simple method 2) is found to be best performing of the simple homology transfer methods; outperforming both single network and MUNK (but never MUNDO). All methods are shown with parameter settings of *c*, *d* and *α*, as noted (full parameter results appear in Supplementary Materials).

**Table 2.** Mean percent accuracy, *F*1-max and Resnik scores (standard dev.) reported for *M.Musculus* → *H.sapiens* over 10 runs of *inverted* (Section 3.5) *k*-fold cross-validation

	DSD	Top blast hit + neighborhood	MUNK	MUNDO
2-Fold				
Accuracy	10.98 ± 0.17	10.65 ± 0.35	8.35 ± 0.05	**11.64 ± 0.20**
*F*1-max	6.48 ± 0.01	6.48 ± 0.06	4.47 ± 0.05	**7.22** ± 0.01
MF Resnik	4.22 ± 0.31	**4.69 ± 0.01**	3.76 ± 0.01	4.41 ± 0.28
BP Resnik	3.81 ± 0.08	3.97 ± 0.26	2.11 ± 0.57	**4.07 ± 0.46**
4-Fold				
Accuracy	9.60 ± 0.17	9.24 ± 0.13	8.34 ± 0.08	**10.94 ± 0.13**
*F*1-max	5.83 ± 0.06	5.86 ± 0.21	4.46 ± 0.03	**6.62 ± 0.02**
MF Resnik	4.18 ± 0.31	4.12 ± 0.62	3.95 ± 0.13	**4.19 ± 0.12**
BP Resnik	**4.48 ± 0.16**	3.32 ± 0.75	2.72 ± 0.27	4.29 ± 0.82
6-Fold				
Accuracy	8.82 ± 0.13	8.52 ± 0.10	8.28 ± 0.04	**10.39 ± 0.10**
*F*1-max	5.34 ± 0.12	5.29 ± 0.14	4.46 ± 0.03	**6.39 ± 0.07**
MF Resnik	4.15 ± 0.42	3.81 ± 0.53	4.00 ± 0.13	**4.32 ± 0.53**
BP Resnik	**4.28 ± 0.84**	3.67 ± 1.56	2.46 ± 0.34	3.89 ± 0.35
10-Fold				
Accuracy	7.79 ± 0.09	7.47 ± 0.13	8.30 ± 0.02	**9.86 ± 0.08**
*F*1-max	4.82 ± 0.14	4.71 ± 0.20	4.45 ± 0.02	**6.08 ± 0.12**
MF Resnik	3.51 ± 1.07	3.31 ± 1.01	**3.97 ± 0.07**	3.78 ± 0.46
BP Resnik	**3.59 ± 1.12**	2.61 ± 1.53	2.51 ± 0.38	3.35 ± 0.54

*Note*: Method settings: For all methods: *d* = 20, *c* = 10, α=1.5. Best performing results are in bold. Additional parameter settings explored in Supplementary Materials.

**Table 3.** Mean percent accuracy, *F*1-max and Resnik scores (standard dev.) reported for *S.cerevisiae* → *S.pombe* over 10 runs of *inverted* (Section 3.5) *k*-fold cross-validation

	DSD	Top blast hit + neighborhood	MUNK	MUNDO
2-Fold				
Accuracy	**25.90 ± 0.83**	20.62 ± 0.52	11.10 ± 0.34	25.14 ± 0.60
*F*1-max	21.01 ± 0.22	15.91 ± 0.13	11.96 ± 0.17	**21.02 ± 0.27**
MF Resnik	1.34 ± 0.10	1.33 ± 0.03	1.29 ± 0.08	**1.90 ± 0.15**
BP Resnik	0.93 ± 0.01	**0.94 ± 0.01**	0.50 ± 0.06	0.93 ± 0.01
4-Fold				
Accuracy	**23.22 ± 0.29**	17.95 ± 0.38	11.09 ± 0.17	22.77 ± 0.30
*F*1-max	**19.15 ± 0.63**	14.17 ± 0.51	11.73 ± 0.19	19.04 ± 0.78
MF Resnik	1.41 ± 0.14	1.41 ± 0.13	1.16 ± 0.02	**1.78 ± 0.30**
BP Resnik	0.87 ± 0.02	**0.89 ± 0.02**	0.56 ± 0.06	0.88 ± 0.01
6-Fold				
Accuracy	**21.94 ± 0.30**	16.06 ± 0.27	11.11 ± 0.09	21.49 ± 0.31
*F*1-max	17.90 ± 0.57	12.77 ± 0.73	11.72 ± 0.15	**18.02 ± 0.66**
MF Resnik	1.25 ± 0.10	1.27 ± 0.09	1.15 ± 0.01	**1.31 ± 0.15**
BP Resnik	0.82 ± 0.04	**0.84 ± 0.04**	0.61 ± 0.06	0.82 ± 0.04
10-Fold				
Accuracy	19.56 ± 0.29	13.64 ± 0.30	11.07 ± 0.07	**19.74 ± 0.27**
*F*1-max	15.93 ± 0.66	10.57 ± 0.47	11.70 ± 0.07	**16.68 ± 0.51**
MF Resnik	1.28 ± 0.11	1.27 ± 0.08	1.14 ± 0.01	**1.50 ± 0.37**
BP Resnik	0.73 ± 0.03	**0.75 ± 0.03**	0.61 ± 0.04	0.73 ± 0.03

*Note*: Method settings: For all methods: *d* = 20, *c* = 10, α=1.5. Best performing results are in bold. Additional parameter settings explored in Supplementary Materials.

**Table 4.** Mean percent accuracy, *F*1-max and Resnik scores (standard dev.) reported for *S.pombe* → *S.cerevisiae* over 10 runs of *inverted* (Section 3.5) *k*-fold cross-validation

	DSD	Top blast hit + neighborhood	MUNK	MUNDO
2-Fold				
Accuracy	8.64 ± 0.31	9.04 ± 0.54	9.39 ± 0.51	**9.62 ± 0.57**
*F*1-max	4.44 ± 0.18	4.46 ± 0.27	3.23 ± 0.05	**4.59 ± 0.01**
MF Resnik	1.06 ± 0.33	**1.09 ± 0.29**	0.55 ± 0.01	0.69 ± 0.01
BP Resnik	0.39 ± 0.02	**0.45 ± 0.02**	0.39 ± 0.02	0.40 ± 0.01
4-Fold				
Accuracy	8.35 ± 0.32	8.43 ± 0.24	9.20 ± 0.15	**9.80 ± 0.41**
*F*1-max	4.54 ± 0.37	4.49 ± 0.08	3.26 ± 0.10	**4.76 ± 0.16**
MF Resnik	0.82 ± 0.25	**0.93 ± 0.25**	0.56 ± 0.01	0.69 ± 0.01
BP Resnik	**0.67 ± 0.43**	0.51 ± 0.15	0.40 ± 0.01	**0.67 ± 0.43**
6-Fold				
Accuracy	8.50 ± 0.24	8.33 ± 0.17	9.30 ± 0.13	**9.93 ± 0.27**
*F*1-max	4.49 ± 0.34	4.44 ± 0.19	3.23 ± 0.09	**4.73 ± 0.20**
MF Resnik	0.72 ± 0.07	0.91 ± 0.27	0.56 ± 0.01	**0.97 ± 0.39**
BP Resnik	0.40 ± 0.08	0.62 ± 0.27	0.40 ± 0.01	**0.41 ± 0.06**
10-Fold				
Accuracy	8.30 ± 0.15	8.25 ± 0.18	9.29 ± 0.05	**9.94 ± 0.13**
*F*1-max	4.46 ± 0.23	4.34 ± 0.24	3.26 ± 0.08	**4.61 ± 0.22**
MF Resnik	0.96 ± 0.46	0.97 ± 0.38	0.56 ± 0.01	**0.98 ± 0.45**
BP Resnik	0.39 ± 0.04	**0.49 ± 0.19**	0.40 ± 0.01	0.39 ± 0.04

*Note*: Method settings: For all methods: *d* = 20, *c* = 10, α=1.5. Best performing results are in bold. Additional parameter settings explored in Supplementary Materials.

### 4.1 Running time

The most computationally expensive step of MUNDO is to compute the single DSD network embedding of the target species, and the combined MUNK embedding of both species. We make the following remarks: (i) the embedding step only has to be done once for a species pair, (ii) we were able to compute both the MUNK and DSD embedding steps for our experiments even using exact, converged DSD, in ∼8 h for the human/mouse embedding, and only a couple of hours for the much smaller yeast/yeast networks on a desktop computer: all others steps take seconds to assign functional labels and (iii) if even the exact DSD and combined embedding computations are considered too expensive, we could instead compute an approximate DSD much faster using methods described in Lin *et al.* (2018).

## 5 Discussion

We have introduced MUNDO, a new co-embedding method that leverages the power of multiple species to improve functional label prediction. We optimized MUNDO for predicting mouse functional labels, when trained on mouse and human PPI networks, but showed that the same parameter settings give good performance when predicting human functional labels (trained on mouse and human) and predicting *S.cerevisiae* labels when trained on both *S.cerevisiae* and *S.pombe*. However, MUNDO did not perform as well as the single-network method in predicting *S.pombe* annotations when also including the *S.cerevisiae* network in the co-embedding, and in fact, performance degraded for all the methods we compared against that tried to incorporate the second species for this case (except for MUNDO when looking at the sparsest number of training labels, where MUNDO actually slightly outperformed the single-network method). It is not clear why that might be, but the most likely explanation is that the *S.cerevisiae* data is somehow noisier or differently structured: we note (Section 3.1) that we included genetic interactions as well as physical interactions in our PPI networks, but *S.cerevisiae* has the largest proportion of genetic interactions, so if they distort the network co-embedding, this could be a possible explanation for the weaker performance. We note that the performance under all three measures we consider largely followed the same trends; in cases with more training data, MUNDO’s Resnik score was sometimes only second best (in these cases, the best performer was split among all the other methods).

We are also interested in applying MUNDO to other PPI networks and other pairs of species, but we note that the limiting factor, for both MUNDO and its predecessor MUNK, is finding pairs of species that are sufficiently evolutionarily close that a robust set of landmarks can be found to accomplish the mapping. Using the proposed RBH method, this is not currently possible, e.g. to map between human and yeast; the species distance is just too great. It is an interesting area of open research to determine if other methods of landmark selection that relax the RBH condition can be learned to map between more distant species in order to pursue an MUNDO approach. One strategy would be to adapt existing algorithms for the global network alignment problem [such as El-Kebir *et al.* (2015), Hashemifar and Xu (2014), Kuchaiev and Przulj (2011), Neyshabur *et al.* (2013), Patro and Kingsford (2012), Sahraeian and Yoon (2013), Singh *et al.* (2008), Vijayan *et al.* (2015), or see Guzzi and Milenković (2018) for a recent survey], most of which construct their overall alignment in two steps; first, they compute a global similarity score, and then they apply some sort of exact or heuristic weighted matching algorithm to produce the correspondence. The most straightforward approach would be to take matched node pairs whose similarity score is above some threshold as the landmark set. We will explore the performance of various global network alignment methods and similarity measurement and matching strategies to produce good landmarks for MUNDO embeddings of more evolutionary distant species in future work.

MUNDO is freely available on the public facing GitHub repository, together with the networks and embeddings discussed in this paper. MUNDO is easy to run on all your favorite PPI networks, since the software currently supports a wide range of popular PPI network formats, including BioGRID, BioPlex, DIP, GeneMANIA, GIANT, HumanNET, Reactome and STRING. Each database has its own disjoint set of protein identifiers called its *namespace*, making it difficult to directly compare one network to another. To allow MUNDO to compare proteins in different namespaces, all protein identifiers are mapped to the RefSeq namespace (Pruitt *et al.*, 2014), which is the format used by NCBI’s BLAST suite. Depending on the database of origin of each species, proteins are either directly mapped to RefSeq from their original format or indirectly mapped to the ubiquitous UniProt (Apweiler *et al.*, 2004) namespace, and then from UniProt to RefSeq. Once all the proteins in both networks have been BLASTed against one another, their original UniProt identifiers are reused to map each node to its appropriate GO labels.

## Supplementary Material

vbab025_Supplementary_Data
