# Supplementary material for: MUNDO: protein function prediction embedded in a multispecies world
Source: Bioinform Adv. 2021 Sep 29;2(1):vbab025. doi: 10.1093/bioadv/vbab025 (PMC9710620; doi:10.1093/bioadv/vbab025)
Supplement: vbab025_Supplementary_Data [file vbab025_supplementary_data.pdf]

MUNDO: Protein Function Prediction Embedded in a Multi-Species World (Supplementary Materials)

Victor Arsenescu Kapil Devkota Mert Erden Polina Shpilker Matthew Werenski Lenore J. Cowen*

Department of Computer Science, Tufts University, Medford, MA, 02155, USA
*To whom correspondence should be addressed. cowen@cs.tufts.edu

1 Parameter Selection

1.1 H. Sapiens (Human) → M. Musculus (Mouse):

As discussed in Section 2.4 of the main paper, MUNDO has five hyperparameters that can be tuned: p, q, c, d, and α, and the competitor methods we test, depending on method, set some or all of the same hyperparameters. Parameters p and q determine the number of landmarks for MUNK and the co-embedding piece of MUNDO; we do not tune these but instead set them as MUNK's authors recommend in their paper (see Discussion in the main paper). We explore how to set the other four parameters in the first of the four species experiments we conduct (namely, human as the source species and mouse as the target species). We randomly split the labeled data 50/50 into a training and validation set, and explore parameter settings on the training set below, in 5-fold cross validation. In order to explore the optimal values of each parameter for each method in this species setting, we first varied the values of d and c while holding α constant at 1 for MUNDO, which we call the *unweighted* results. (Note that only the competitor methods listed with a "+ neighbors" set both c and d parameters: i.e. DSD and the BLAST methods that are not listed with a "+ Neighbors", only depend on d, not c, while MUNK only sets c, not d.) We performed a grid search over all combinations of (d, c), where the values of d and c were chosen independently from the set $\{5, 10, 20\}$ for MUNDO and all competitor methods in our 5-fold cross validation experiment over our training set. Results appear in Table S1. We note that setting $d = 20$ for the size of the neighborhood in the target species was superior to $d = 5$ and $d = 10$ for all methods we tested. In addition, MUNDO dominates all the other methods at all parameter settings.

Next we explored how to set α for MUNDO. Based on the unweighted results from Table S1, we fixed the values of d to 20. We performed another grid search, choosing α from the set $\{1.0, 1.5, 2.0\}$. Based on the results in Tables S2 - S4, we set $d = 20, c = 10$, and $\alpha = 1.5$ for MUNDO in the main paper. Furthermore, we kept these values for MUNDO's parameters fixed in the other three species experiments in the main paper (but explore other parameter settings in the supplemental tables in Section 2 below.

		(5, 5)	(5, 10)	(5, 20)	(10, 5)	(10, 10)	(10, 20)	(20, 5)	(20, 10)	(20, 20)
**DSD**	Accuracy	11.34 ± 0.23	11.34 ± 0.23	11.34 ± 0.23	12.54 ± 0.29	12.54 ± 0.29	12.54 ± 0.29	13.34 ± 0.63	13.34 ± 0.63	13.34 ± 0.63
	F1-max	7.64 ± 0.40	7.64 ± 0.40	7.64 ± 0.40	8.74 ± 0.12	8.74 ± 0.12	8.74 ± 0.12	9.66 ± 0.35	9.66 ± 0.35	9.66 ± 0.35
**Top Blast Hit**	Accuracy	12.20 ± 0.68	12.20 ± 0.68	12.20 ± 0.68	13.77 ± 0.52	13.77 ± 0.52	13.77 ± 0.52	14.12 ± 0.41	14.12 ± 0.41	14.12 ± 0.41
	F1-max	8.74 ± 0.27	8.74 ± 0.27	8.74 ± 0.27	9.59 ± 0.35	9.59 ± 0.35	9.59 ± 0.35	10.17 ± 0.30	10.17 ± 0.30	**10.17 ± 0.30**
**Top Blast Hit**	Accuracy	11.98 ± 0.25	11.40 ± 0.32	10.47 ± 0.27	13.68 ± 0.64	12.98 ± 0.72	11.88 ± 0.65	14.39 ± 0.48	14.21 ± 0.36	13.26 ± 0.32
**+ Neighbors**	F1-max	8.55 ± 0.26	8.11 ± 0.23	7.48 ± 0.31	9.61 ± 0.31	9.18 ± 0.28	8.56 ± 0.20	10.27 ± 0.32	10.04 ± 0.30	9.59 ± 0.29
**All Blast Hits**	Accuracy	10.62 ± 0.46	10.62 ± 0.46	10.62 ± 0.46	12.56 ± 0.48	12.56 ± 0.48	12.56 ± 0.48	13.43 ± 0.27	13.43 ± 0.27	13.43 ± 0.27
	F1-max	7.62 ± 0.24	7.62 ± 0.24	7.62 ± 0.24	8.90 ± 0.35	8.90 ± 0.35	8.90 ± 0.35	9.60 ± 0.18	9.60 ± 0.18	9.60 ± 0.18
**All Blast Hits**	Accuracy	9.65 ± 0.34	8.89 ± 0.30	8.34 ± 0.37	11.75 ± 0.68	11.10 ± 0.35	10.14 ± 0.61	13.08 ± 0.43	12.45 ± 0.29	11.77 ± 0.26
**+ Neighbors**	F1-max	7.03 ± 0.27	6.66 ± 0.30	6.23 ± 0.47	8.41 ± 0.26	8.17 ± 0.24	7.59 ± 0.24	9.42 ± 0.28	9.17 ± 0.30	8.77 ± 0.33
**Thresholded Blast Hits**	Accuracy	11.19 ± 0.40	11.19 ± 0.40	11.19 ± 0.40	12.86 ± 0.51	12.86 ± 0.51	12.86 ± 0.51	13.60 ± 0.44	13.60 ± 0.44	13.60 ± 0.44
	F1-max	8.03 ± 0.30	8.03 ± 0.30	8.03 ± 0.30	9.04 ± 0.27	9.04 ± 0.27	9.04 ± 0.27	9.77 ± 0.22	9.77 ± 0.22	9.77 ± 0.22
**Thresholded Blast Hits**	Accuracy	11.14 ± 0.33	11.04 ± 0.33	10.78 ± 0.33	12.77 ± 0.46	12.72 ± 0.45	12.49 ± 0.43	13.62 ± 0.46	13.66 ± 0.51	13.48 ± 0.49
**+ Neighbors**	F1-max	8.06 ± 0.34	7.96 ± 0.30	7.86 ± 0.26	9.13 ± 0.29	9.08 ± 0.27	9.01 ± 0.27	9.78 ± 0.20	9.83 ± 0.20	9.75 ± 0.17
**MUNK**	Accuracy	11.27 ± 0.44	11.83 ± 0.70	12.37 ± 0.76	11.27 ± 0.44	11.83 ± 0.70	12.37 ± 0.76	11.27 ± 0.44	11.83 ± 0.70	12.37 ± 0.76
	F1-max	7.74 ± 0.30	8.59 ± 0.30	8.74 ± 0.46	7.74 ± 0.30	8.59 ± 0.30	8.74 ± 0.46	7.74 ± 0.30	8.59 ± 0.30	8.74 ± 0.46
**MUNDO**	Accuracy	**14.54 ± 0.24**	**14.62 ± 0.90**	**13.87 ± 0.95**	**14.93 ± 0.89**	**15.21 ± 0.78**	**14.53 ± 0.78**	**15.91 ± 0.83**	**15.66 ± 0.61**	**15.15 ± 0.85**
	F1-max	**9.91 ± 0.31**	**9.81 ± 0.47**	**9.65 ± 0.41**	**10.33 ± 0.33**	**10.26 ± 0.57**	**9.80 ± 0.42**	**10.88 ± 0.38**	**10.83 ± 0.51**	10.00 ± 0.49

Table S1: H. Sapiens → M. Musculus MUNDO Results: Unweighted Grid Search over all values of (d, c) with $(q, p) = (90\%, 85\%)$

	1.00	1.50	2.00
Accuracy	15.91 ± 0.83	**16.00 ± 0.83**	15.39 ± 0.94
F1-max	**10.88 ± 0.38**	10.80 ± 0.44	10.63 ± 0.53

Table S2: H. Sapiens → M. Musculus MUNDO Results: Varying α with $(q, p) = (90\%, 85\%)$, $(d, c) = (20, 5)$

	1.00	1.50	2.00
Accuracy	15.66 ± 0.61	**16.18 ± 0.55**	16.17 ± 0.74
F1-max	10.83 ± 0.51	**11.02 ± 0.51**	11.01 ± 0.37

Table S3: H. Sapiens → M. Musculus MUNDO Results: varying α with $(q, p) = (90\%, 85\%)$, $(d, c) = (20, 10)$

	1.00	1.50	2.00
Accuracy	15.91 ± 0.83	**16.00 ± 0.83**	15.39 ± 0.94
F1-max	**10.88 ± 0.38**	10.80 ± 0.44	10.63 ± 0.53

Table S4: H. Sapiens → M. Musculus MUNDO Results: Varying α with $(q, p) = (90\%, 85\%)$, $(d, c) = (20, 20)$

2 M. Musculus (Mouse) $\rightarrow$ H. Sapiens (Human):

In the main paper, we focus on the recommended default settings from the human to mouse settings in all the other species mapping experiments. However, the next table explores explores the percent accuracy we would obtained in our inverted cross-validation experiments when embedding the original mouse and human networks in the opposite direction: that is, making the mouse network the *model* network and making the human network the *target* network for the three core methods: DSD, MUNK, and MUNDO. We keep α, p, and q as recommended in the first species experiment for MUNDO, but vary our choice of c and d. MUNDO's performance dominates DSD and MUNK over all parameter choices we tested.

		**(5, 5)**	**(5, 10)**	**(5, 20)**	**(10, 5)**	**(10, 10)**	**(10, 20)**	**(20, 5)**	**(20, 10)**	**(20, 20)**
**DSD**	4-fold Accuracy	6.13 ± 0.20	7.18 ± 0.03	7.98 ± 0.16	6.13 ± 0.20	7.18 ± 0.03	7.98 ± 0.16	6.13 ± 0.20	7.18 ± 0.03	7.98 ± 0.16
	4-fold F1-max	4.53 ± 0.14	5.24 ± 0.09	5.99 ± 0.04	4.53 ± 0.14	5.24 ± 0.09	5.99 ± 0.04	4.53 ± 0.14	5.24 ± 0.09	5.99 ± 0.04
	6-fold Accuracy	5.61 ± 0.21	6.58 ± 0.26	7.37 ± 0.28	5.61 ± 0.21	6.58 ± 0.26	7.37 ± 0.28	5.61 ± 0.21	6.58 ± 0.26	7.37 ± 0.28
	6-fold F1-max	4.17 ± 0.07	4.86 ± 0.13	5.51 ± 0.17	4.17 ± 0.07	4.86 ± 0.13	5.51 ± 0.17	4.17 ± 0.07	4.86 ± 0.13	5.51 ± 0.17
**MUNK**	4-fold Accuracy	6.81 ± 0.03	6.81 ± 0.03	6.81 ± 0.03	6.89 ± 0.10	6.89 ± 0.10	6.89 ± 0.10	7.00 ± 0.03	7.00 ± 0.03	7.00 ± 0.03
	4-fold F1-max	4.52 ± 0.05	4.52 ± 0.05	4.52 ± 0.05	4.85 ± 0.03	4.85 ± 0.03	4.85 ± 0.03	5.15 ± 0.04	5.15 ± 0.04	5.15 ± 0.04
	6-fold Accuracy	6.85 ± 0.04	6.85 ± 0.04	6.85 ± 0.04	6.93 ± 0.07	6.93 ± 0.07	6.93 ± 0.07	6.99 ± 0.07	6.99 ± 0.07	6.99 ± 0.07
	6-fold F1-max	4.51 ± 0.04	4.51 ± 0.04	4.51 ± 0.04	4.86 ± 0.03	4.86 ± 0.03	4.86 ± 0.03	5.13 ± 0.06	5.13 ± 0.06	5.13 ± 0.06
**MUNDO**	4-fold Accuracy	**8.28 ± 0.12**	**8.65 ± 0.10**	**8.98 ± 0.14**	**8.14 ± 0.17**	**8.48 ± 0.17**	**8.99 ± 0.05**	**7.82 ± 0.17**	**8.21 ± 0.17**	**8.73 ± 0.23**
	4-fold F1-max	**6.02 ± 0.06**	**6.37 ± 0.07**	**6.77 ± 0.05**	**6.02 ± 0.07**	**6.36 ± 0.03**	**6.73 ± 0.06**	**5.92 ± 0.08**	**6.29 ± 0.04**	**6.60 ± 0.05**
	6-fold Accuracy	**8.04 ± 0.17**	**8.30 ± 0.20**	**8.65 ± 0.33**	**7.98 ± 0.24**	**8.31 ± 0.16**	**8.72 ± 0.23**	**7.64 ± 0.11**	**7.99 ± 0.16**	**8.47 ± 0.18**
	6-fold F1-max	**5.89 ± 0.07**	**6.14 ± 0.12**	**6.38 ± 0.16**	**5.85 ± 0.08**	**6.15 ± 0.06**	**6.42 ± 0.08**	**5.77 ± 0.07**	**6.02 ± 0.07**	**6.31 ± 0.07**

Table S5: M. Musculus $\rightarrow$ H. Sapiens Weighted Grid Search over all values of (d, c) with $(q, p) = (90\%, 85\%)$, $\alpha = 1.5$

3 S. pombe (Fission Yeast) $\rightarrow$ S. cerevisiae (Baker's Yeast): Setting RBH Thresholds

In this section, we explore different settings for the parameters p and q that provide a reasonable number of reciprocal best BLAST hits to use as landmarks in two new species: *S. cerevisiae* and *S. pombe*, at the same time as we explore different choices for the d and c parameters. As discussed in Section 3.1 of the original paper, the smaller yeast networks are roughly one fifth the size of the larger human network. We chose thresholds such that we would have at least 60 landmarks. Table S6 gives results setting $(q, p) = (75\%, 50\%)$, and Table S7 gives results setting $(q, p) = (50\%, 50\%)$.

		**(5, 5)**	**(5, 10)**	**(5, 20)**	**(10, 5)**	**(10, 10)**	**(10, 20)**	**(20, 5)**	**(20, 10)**	**(20, 20)**
**DSD**	4-fold Accuracy	11.64 ± 0.28	11.76 ± 0.25	12.05 ± 0.29	11.64 ± 0.28	11.76 ± 0.25	12.05 ± 0.29	**11.64 ± 0.28**	11.76 ± 0.25	12.05 ± 0.29
	4-fold F1-max	13.13 ± 0.29	13.36 ± 0.32	13.57 ± 0.27	13.13 ± 0.29	13.36 ± 0.32	13.57 ± 0.27	**13.13 ± 0.29**	13.36 ± 0.32	13.57 ± 0.27
	6-fold Accuracy	10.11 ± 0.17	10.37 ± 0.30	10.56 ± 0.35	10.11 ± 0.17	10.37 ± 0.30	10.56 ± 0.35	10.11 ± 0.17	10.37 ± 0.30	10.56 ± 0.35
	6-fold F1-max	11.66 ± 0.38	12.13 ± 0.36	12.21 ± 0.31	11.66 ± 0.38	12.13 ± 0.36	12.21 ± 0.31	11.66 ± 0.38	12.13 ± 0.36	12.21 ± 0.31
**MUNK**	4-fold Accuracy	6.75 ± 0.08	6.75 ± 0.08	6.75 ± 0.08	7.86 ± 0.09	7.86 ± 0.09	7.86 ± 0.09	7.36 ± 0.15	7.36 ± 0.15	7.36 ± 0.15
	4-fold F1-max	7.33 ± 0.04	7.33 ± 0.04	7.33 ± 0.04	8.40 ± 0.18	8.40 ± 0.18	8.40 ± 0.18	7.93 ± 0.16	7.93 ± 0.16	7.93 ± 0.16
	6-fold Accuracy	6.74 ± 0.12	6.74 ± 0.12	6.74 ± 0.12	7.88 ± 0.16	7.88 ± 0.16	7.88 ± 0.16	7.43 ± 0.18	7.43 ± 0.18	7.43 ± 0.18
	6-fold F1-max	7.41 ± 0.12	7.41 ± 0.12	7.41 ± 0.12	8.48 ± 0.21	8.48 ± 0.21	8.48 ± 0.21	7.96 ± 0.17	7.96 ± 0.17	7.96 ± 0.17
**MUNDO**	4-fold Accuracy	**12.06 ± 0.23**	**12.34 ± 0.17**	**12.33 ± 0.27**	**12.04 ± 0.21**	**12.47 ± 0.22**	**12.42 ± 0.25**	11.48 ± 0.05	**12.16 ± 0.18**	**12.29 ± 0.26**
	4-fold F1-max	**13.54 ± 0.32**	**13.88 ± 0.18**	**13.79 ± 0.25**	**13.86 ± 0.23**	**13.85 ± 0.22**	**13.99 ± 0.16**	12.98 ± 0.21	**13.63 ± 0.11**	**13.92 ± 0.22**
	6-fold Accuracy	**10.89 ± 0.15**	**11.11 ± 0.30**	**10.95 ± 0.37**	**11.23 ± 0.38**	**11.38 ± 0.41**	**11.25 ± 0.35**	**10.66 ± 0.44**	**11.31 ± 0.47**	**11.32 ± 0.40**
	6-fold F1-max	**12.40 ± 0.40**	**12.68 ± 0.32**	**12.63 ± 0.19**	**12.83 ± 0.43**	**12.86 ± 0.33**	**12.85 ± 0.23**	**12.12 ± 0.32**	**12.60 ± 0.36**	**12.82 ± 0.19**

Table S6: S. pombe $\rightarrow$ S. cerevisiae Weighted Grid Search over all values of (d, c) with $(q, p) = (75\%, 50\%)$, $\alpha = 1.5$

		**(5, 5)**	**(5, 10)**	**(5, 20)**	**(10, 5)**	**(10, 10)**	**(10, 20)**	**(20, 5)**	**(20, 10)**	**(20, 20)**
**DSD**	4-fold Accuracy	11.64 ± 0.28	11.76 ± 0.25	12.05 ± 0.29	11.64 ± 0.28	11.76 ± 0.25	12.05 ± 0.29	**11.64 ± 0.28**	11.76 ± 0.25	12.05 ± 0.29
	4-fold F1-max	13.13 ± 0.29	13.36 ± 0.32	13.57 ± 0.27	13.13 ± 0.29	13.36 ± 0.32	13.57 ± 0.27	**13.13 ± 0.29**	13.36 ± 0.32	13.57 ± 0.27
	6-fold Accuracy	10.11 ± 0.17	10.37 ± 0.30	10.56 ± 0.35	10.11 ± 0.17	10.37 ± 0.30	10.56 ± 0.35	10.11 ± 0.17	10.37 ± 0.30	10.56 ± 0.35
	6-fold F1-max	11.66 ± 0.38	12.13 ± 0.36	12.21 ± 0.31	11.66 ± 0.38	12.13 ± 0.36	12.21 ± 0.31	11.66 ± 0.38	12.13 ± 0.36	12.21 ± 0.31
**MUNK**	4-fold Accuracy	7.43 ± 0.13	7.43 ± 0.13	7.43 ± 0.13	7.52 ± 0.14	7.52 ± 0.14	7.52 ± 0.14	7.24 ± 0.14	7.24 ± 0.14	7.24 ± 0.14
	4-fold F1-max	8.01 ± 0.13	8.01 ± 0.13	8.01 ± 0.13	9.47 ± 0.18	9.47 ± 0.18	9.47 ± 0.18	8.28 ± 0.15	8.28 ± 0.15	8.28 ± 0.15
	6-fold Accuracy	7.42 ± 0.13	7.42 ± 0.13	7.42 ± 0.13	7.58 ± 0.15	7.58 ± 0.15	7.58 ± 0.15	7.28 ± 0.21	7.28 ± 0.21	7.28 ± 0.21
	6-fold F1-max	8.08 ± 0.15	8.08 ± 0.15	8.08 ± 0.15	9.59 ± 0.20	9.59 ± 0.20	9.59 ± 0.20	8.38 ± 0.26	8.38 ± 0.26	8.38 ± 0.26
**MUNDO**	4-fold Accuracy	**12.19 ± 0.32**	**12.55 ± 0.16**	**12.50 ± 0.10**	**12.12 ± 0.25**	**12.42 ± 0.20**	**12.61 ± 0.20**	11.59 ± 0.18	**12.16 ± 0.24**	**12.42 ± 0.21**
	4-fold F1-max	**13.77 ± 0.30**	**13.98 ± 0.21**	**13.98 ± 0.27**	**13.90 ± 0.30**	**14.03 ± 0.20**	**14.05 ± 0.14**	13.10 ± 0.27	**13.66 ± 0.20**	**14.02 ± 0.19**
	6-fold Accuracy	**11.16 ± 0.13**	**11.23 ± 0.40**	**11.12 ± 0.38**	**11.27 ± 0.31**	**11.43 ± 0.45**	**11.29 ± 0.40**	**10.80 ± 0.39**	**11.36 ± 0.51**	**11.32 ± 0.37**
	6-fold F1-max	**12.71 ± 0.38**	**12.92 ± 0.36**	**12.86 ± 0.21**	**13.09 ± 0.44**	**13.07 ± 0.40**	**13.01 ± 0.23**	**12.20 ± 0.37**	**12.79 ± 0.45**	**13.00 ± 0.22**

Table S7: S. pombe $\rightarrow$ S. cerevisiae Weighted Grid Search over all values of (d, c) with $(q, p) = (50\%, 50\%)$, $\alpha = 1.5$

4 S. cerevisiae (Baker's Yeast) → S. pombe (Fission Yeast) : Setting RBH Thresholds

In this section, we explore different settings for the parameters p and q that provide a reasonable number of reciprocal best BLAST hits to use as landmarks when embedding the two yeast networks in the opposite direction: that is, making S. cerevisiae the *model* network and making S. pombe the *target* network for the three core methods: DSD, MUNK, and MUNDO. We keep α as recommended in the first species experiment for MUNDO, but vary our choice of d and c. As discussed in Section 3.1 of the original paper, the smaller yeast networks are roughly one fifth the size of the larger human network. We chose thresholds such that we would have at least 60 landmarks. Table S8 gives results setting $(q,p) = (75\%, 50\%)$, and Table S9 gives results setting $(q,p) = (50\%, 50\%)$.

		**(5, 5)**	**(5, 10)**	**(5, 20)**	**(10, 5)**	**(10, 10)**	**(10, 20)**	**(20, 5)**	**(20, 10)**	**(20, 20)**
**DSD**	4-fold Accuracy	**8.42 ± 0.49**	8.02 ± 0.48	7.80 ± 0.46	**8.42 ± 0.49**	**8.02 ± 0.48**	7.80 ± 0.46	**8.42 ± 0.49**	**8.02 ± 0.48**	**7.80 ± 0.46**
	4-fold F1-max	12.05 ± 0.47	11.70 ± 0.71	11.56 ± 0.33	**12.05 ± 0.47**	11.70 ± 0.71	11.56 ± 0.33	**12.05 ± 0.47**	11.70 ± 0.71	11.56 ± 0.33
	6-fold Accuracy	7.94 ± 0.49	7.64 ± 0.50	7.05 ± 0.43	**7.94 ± 0.49**	7.64 ± 0.50	7.05 ± 0.43	**7.94 ± 0.49**	**7.64 ± 0.50**	7.05 ± 0.43
	6-fold F1-max	11.32 ± 0.67	10.95 ± 0.62	10.58 ± 0.71	11.32 ± 0.67	10.95 ± 0.62	10.58 ± 0.71	11.32 ± 0.67	10.95 ± 0.62	10.58 ± 0.71
**MUNK**	4-fold Accuracy	5.34 ± 0.18	5.34 ± 0.18	5.34 ± 0.18	5.38 ± 0.13	5.38 ± 0.13	5.38 ± 0.13	5.39 ± 0.31	5.39 ± 0.31	5.39 ± 0.31
	4-fold F1-max	7.88 ± 0.19	7.88 ± 0.19	7.88 ± 0.19	8.53 ± 0.14	8.53 ± 0.14	8.53 ± 0.14	10.04 ± 0.17	10.04 ± 0.17	10.04 ± 0.17
	6-fold Accuracy	5.44 ± 0.11	5.44 ± 0.11	5.44 ± 0.11	5.47 ± 0.15	5.47 ± 0.15	5.47 ± 0.15	5.47 ± 0.14	5.47 ± 0.14	5.47 ± 0.14
	6-fold F1-max	8.04 ± 0.11	8.04 ± 0.11	8.04 ± 0.11	8.74 ± 0.22	8.74 ± 0.22	8.74 ± 0.22	10.17 ± 0.26	10.17 ± 0.26	10.17 ± 0.26
**MUNDO**	4-fold Accuracy	8.41 ± 0.35	**8.49 ± 0.31**	**8.25 ± 0.33**	7.54 ± 0.40	7.94 ± 0.44	**7.98 ± 0.32**	6.60 ± 0.35	7.33 ± 0.43	7.60 ± 0.23
	4-fold F1-max	**12.33 ± 0.34**	**12.75 ± 0.44**	**12.53 ± 0.36**	11.65 ± 0.49	**12.89 ± 0.39**	**12.97 ± 0.51**	11.88 ± 0.42	**13.27 ± 0.56**	**13.48 ± 0.69**
	6-fold Accuracy	**8.21 ± 0.40**	**8.21 ± 0.38**	**7.80 ± 0.51**	7.47 ± 0.44	**7.85 ± 0.41**	**7.68 ± 0.44**	6.56 ± 0.23	7.02 ± 0.23	**7.39 ± 0.42**
	6-fold F1-max	**12.09 ± 0.63**	**12.14 ± 0.42**	**11.65 ± 0.46**	**11.33 ± 0.46**	**12.07 ± 0.31**	**11.84 ± 0.26**	**11.80 ± 0.32**	**12.58 ± 0.39**	**12.58 ± 0.61**

Table S8: S. cerevisiae → S. pombe Weighted Grid Search over all values of (d, c) with $(q,p) = (75\%, 50\%)$, $\alpha = 1.5$

		**(5, 5)**	**(5, 10)**	**(5, 20)**	**(10, 5)**	**(10, 10)**	**(10, 20)**	**(20, 5)**	**(20, 10)**	**(20, 20)**
**DSD**	4-fold Accuracy	8.42 ± 0.49	8.02 ± 0.48	7.80 ± 0.46	**8.42 ± 0.49**	8.02 ± 0.48	7.80 ± 0.46	**8.42 ± 0.49**	**8.02 ± 0.48**	7.80 ± 0.46
	4-fold F1-max	12.05 ± 0.47	11.70 ± 0.71	11.56 ± 0.33	**12.05 ± 0.47**	11.70 ± 0.71	11.56 ± 0.33	12.05 ± 0.47	11.70 ± 0.71	11.56 ± 0.33
	6-fold Accuracy	7.94 ± 0.49	7.64 ± 0.50	7.05 ± 0.43	**7.94 ± 0.49**	7.64 ± 0.50	7.05 ± 0.43	**7.94 ± 0.49**	**7.64 ± 0.50**	7.05 ± 0.43
	6-fold F1-max	11.32 ± 0.67	10.95 ± 0.62	10.58 ± 0.71	11.32 ± 0.67	10.95 ± 0.62	10.58 ± 0.71	11.32 ± 0.67	10.95 ± 0.62	10.58 ± 0.71
**MUNK**	4-fold Accuracy	5.56 ± 0.20	5.56 ± 0.20	5.56 ± 0.20	5.50 ± 0.24	5.50 ± 0.24	5.50 ± 0.24	5.46 ± 0.31	5.46 ± 0.31	5.46 ± 0.31
	4-fold F1-max	8.05 ± 0.21	8.05 ± 0.21	8.05 ± 0.21	8.75 ± 0.23	8.75 ± 0.23	8.75 ± 0.23	10.25 ± 0.27	10.25 ± 0.27	10.25 ± 0.27
	6-fold Accuracy	5.67 ± 0.10	5.67 ± 0.10	5.67 ± 0.10	5.58 ± 0.12	5.58 ± 0.12	5.58 ± 0.12	5.59 ± 0.14	5.59 ± 0.14	5.59 ± 0.14
	6-fold F1-max	8.18 ± 0.17	8.18 ± 0.17	8.18 ± 0.17	8.86 ± 0.14	8.86 ± 0.14	8.86 ± 0.14	10.40 ± 0.28	10.40 ± 0.28	10.40 ± 0.28
**MUNDO**	4-fold Accuracy	**8.53 ± 0.34**	**8.61 ± 0.33**	**8.29 ± 0.33**	7.76 ± 0.43	**8.10 ± 0.47**	**8.06 ± 0.29**	6.88 ± 0.45	7.40 ± 0.57	**7.85 ± 0.23**
	4-fold F1-max	**12.26 ± 0.36**	**12.70 ± 0.54**	**12.47 ± 0.36**	11.91 ± 0.60	**13.00 ± 0.62**	**13.04 ± 0.58**	**12.16 ± 0.61**	**13.38 ± 0.64**	**13.44 ± 0.65**
	6-fold Accuracy	**8.34 ± 0.44**	**8.21 ± 0.41**	**7.74 ± 0.50**	7.65 ± 0.42	**7.92 ± 0.30**	**7.84 ± 0.36**	6.75 ± 0.26	7.47 ± 0.23	**7.65 ± 0.37**
	6-fold F1-max	**12.13 ± 0.52**	**12.04 ± 0.34**	**11.59 ± 0.48**	**11.53 ± 0.38**	**12.24 ± 0.33**	**12.02 ± 0.51**	**12.00 ± 0.51**	**12.62 ± 0.54**	**12.71 ± 0.52**

Table S9: S. cerevisiae → S. pombe Weighted Grid Search over all values of (d, c) with $(q,p) = (50\%, 50\%)$, $\alpha = 1.5$
